# Differential Induction of TLR3-Dependent Innate Immune Signaling by Closely Related Parasite Species

**DOI:** 10.1371/journal.pone.0088398

**Published:** 2014-02-05

**Authors:** Daniel P. Beiting, Lucia Peixoto, Natalia S. Akopyants, Stephen M. Beverley, E. John Wherry, David A. Christian, Christopher A. Hunter, Igor E. Brodsky, David S. Roos

**Affiliations:** 1 Department of Biology, University of Pennsylvania, Philadelphia, Pennsylvania, United States of America; 2 Department of Microbiology, University of Pennsylvania, Philadelphia, Pennsylvania, United States of America; 3 Department of Pathobiology, University of Pennsylvania, Philadelphia, Pennsylvania, United States of America; 4 Department of Molecular Microbiology, Washington University, St. Louis, Missouri, United States of America; University at Buffalo, United States of America

## Abstract

The closely related protozoan parasites *Toxoplasma gondii* and *Neospora caninum* display similar life cycles, subcellular ultrastructure, invasion mechanisms, metabolic pathways, and genome organization, but differ in their host range and disease pathogenesis. Type II (γ) interferon has long been known to be the major mediator of innate and adaptive immunity to *Toxoplasma* infection, but genome-wide expression profiling of infected host cells indicates that *Neospora* is a potent activator of the type I (α/β) interferon pathways typically associated with antiviral responses. Infection of macrophages from mice with targeted deletions in various innate sensing genes demonstrates that host responses to *Neospora* are dependent on the toll-like receptor *Tlr3* and the adapter protein *Trif*. Consistent with this observation, RNA from *Neospora* elicits TLR3-dependent type I interferon responses when targeted to the host endo-lysosomal system. Although live *Toxoplasma* fail to induce type I interferon, heat-killed parasites do trigger this response, albeit much weaker than *Neospora*, and co-infection studies reveal that *T. gondii* actively suppresses the production of type I interferon. These findings reveal that eukaryotic pathogens can be potent inducers of type I interferon and that related parasite species interact with this pathway in distinct ways.

## Introduction

The eukaryotic phylum apicomplexa is comprised of over 5000 species of parasitic protozoa that infect a wide range of animal hosts and cause significant disease in both healthy and immune-compromised individuals. This phylum includes *Plasmodium*, the causative agent of malaria; *Cryptosporidium*, recently recognized to be a leading cause of pediatric diarrheal disease in the developing world [1]; and *Toxoplasma*, a ubiquitous parasite that causes potentially fatal congenital disease and represents the second leading cause of food borne mortality in the USA [2]. Other apicomplexa, including *Babesia, Neospora* and *Theileria* are important pathogens of cattle, while *Eimeria* species are a leading concern in the poultry industry. Despite being important causes of human and animal disease worldwide, there is relatively little information about how these pathogens are recognized by the host innate immune system following invasion of target cells, nor is it known whether infections with distinct protozoa trigger identical, partially overlapping or completely distinct innate immune responses.

The profound susceptibility of IFN-γ-deficient mice to a wide range of protozoan parasites, including *Toxoplasma gondii* [3], [4], *Cryptosporidium parvum* [5], *Leishmania major* [6] and *Trypanosoma cruzi* [7], has given rise to the widely-held view that type II interferon (IFN-γ) is responsible for controlling protozoan infections, in contrast to type I interferons (IFN-α/β) which are primarily associated with control of viral infections. As a consequence, the vast majority of studies on innate immunity to protozoan parasites have focused on IFN-γ produced during acute infection by NK cells [8], [9], and interleukin-12 produced by macrophages [10], neutrophils [11], [12] and dendritic cells [13]. It is unclear whether this accurately captures the complete picture of innate immune pathways triggered by infection with protozoa. Moreover, while tremendous progress has been made in identifying viral and bacterial ligands recognized by innate pattern recognition receptors [14], relatively few studies have explored the mechanisms and consequences of innate recognition of intracellular eukaryotic microbes [15], in part because the conserved signatures of bacteria and viruses are not generally thought to be present in eukaryotic cells.

We have employed a comparative genomic approach to investigate innate immune signaling triggered by two closely related but distinct apicomplexan species, *Toxoplasma gondii* and *Neospora caninum*, both of which are obligate intracellular parasites that initiate infection in the gastrointestinal tract of animals. *Neospora* was only recently recognized as a distinct protozoan species [16], and although it is thought to have diverged from *Toxoplasma* ∼28 million years ago, the two species share nearly indistinguishable ultrastructure [17], metabolism, and 1:1 syntenic orthologs for >90% of the parasite genome [18]. Despite these similarities, important biological differences distinguish the two species with respect to virulence factors, host range, and pathogenesis. *Toxoplasma* undergoes sexual reproduction in felidae (cats) and asexual reproduction in virtually any mammalian host, while the *Neospora* sexual cycle takes place in canidae (dogs), where it demonstrates a propensity for vertical transmission from mother to fetus [19], and infects a more limited range of intermediate hosts [20], [21]. Genomic and transcriptomic analysis of these two parasites reveals that *Neospora* and *Toxoplasma* have evolved distinct repertoires of surface antigens and secreted kinases since divergence from a common ancestor [18], suggesting the ability modulate or interact with host cells in unique ways. We capitalize on the close genetic and ecological relationship between these two parasites to carry out comparative profiling of the host responses to infection. Our results demonstrate that *Neospora* is a potent inducer of innate IFN-α/β responses, and that *Toxoplasma* has evolved the capacity to suppress this response.

## Results

### Differential induction of innate immune signaling by genetically similar parasite species

**Figure 1:**
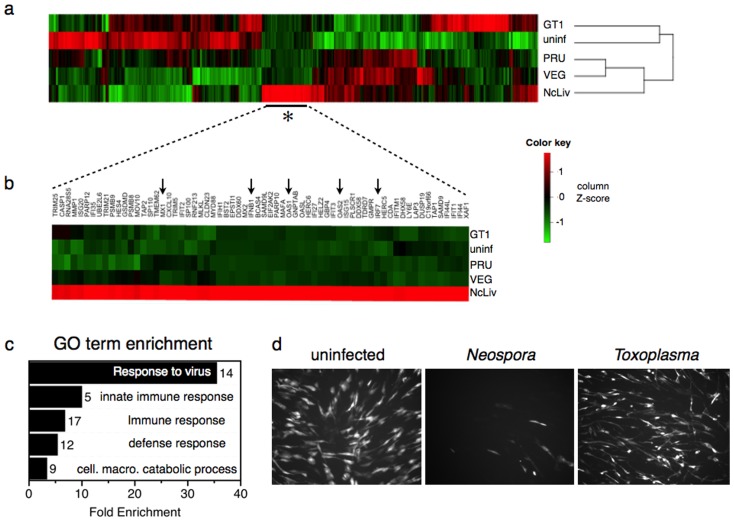
Differential induction of innate immune signaling by genetically related parasite species. a) Microarray-based expression profiling of human foreskin fibroblasts (HFF) infected with *Neospora caninum* (NcLiv isolate) or *Toxoplasma* (GT1, Prugniaud or VEG strains). Heat map shows hierarchical clustering analysis of 822 genes differentially regulated relative to uninfected cells by ≥2-fold (FDR≤5%), in any of these experiments. Each row in heatmap represents average of duplicate (NcLiv and GT1) or triplicate arrays (VEG, PRU, uninf). Color pattern on heatmaps represents column Z-score. b) A cluster of 66 genes (Fig. 1a, asterisk) induced by *Neospora* but not any strain of *Toxoplasma*, including several well-known type I interferon response genes (arrows). c) Gene Ontology (GO) enrichment analysis of 66 *Neospora*-induced genes. Bar graph shows fold enrichment for top five GO Biological Process terms enriched at *P*≤0.05 and represented by ≥5 genes. Number of genes and GO term name is shown at the right of each bar. d) Fluorescence micrographs of uninfected HFF cells, or cells infected with *Neospora* or *Toxoplasma*, then challenged with GFP-tagged vesicular stomatitis virus. Representative images are shown. Experiment was repeated three times with similar results.

In order to compare host transcriptional responses during *Neospora* and *Toxoplasma* infection, whole-genome expression profiling was carried out using human fibroblasts infected with either *Neospora caninum* (NcLiv strain) or *Toxoplasma gondii* (GT1, Prugniaud, or VEG strains, selected as representatives of the three dominant genotypes observed in North America [22], [23]). 822 host genes were identified as differentially transcribed (≥2-fold, FDR≤5%) during infection with any of these four parasites relative to uninfected host cells (Fig. 1a and Table S1). Grouping parasites based on the host response indicated that *Toxoplasma* strains known to exhibit low virulence in mice (PRU, VEG) elicit host response profiles more similar to each other than to the virulent GT1 strain. *Neospora* induced a host response distinct from all strains of *Toxoplasma*, but more closely resembling low-virulence *Toxoplasma* strains, indicating that these two parasite species perturb the host transcriptome in distinct ways.

Hierarchical clustering of the 822 genes revealed a distinctive set of 66 genes induced only during *Neospora* infection (Fig. 1a, asterisk; Fig. 1b), and Gene Ontology (GO) analysis [24] indicated that these are significantly associated with antiviral responses (Fig. 1c). Antiviral genes specifically induced by *Neospora* infection included interferon-β1 (*Ifnb1*) as well as several key regulators of type I interferon responses (Fig. 1b, arrows) [25]–[27], including the transcription factor *Irf7* (a master regulator of the antiviral program), *Oas1* and *Oas2* (members of the 2′-5′-oligoadenylate synthetase family that activate RNase L to initiate degradation of cellular RNA as an innate defense mechanism during viral infection), and *Mx1* (a GTP-binding protein that is essential for antiviral responses to influenza infection). To test whether the transcriptional program induced by *Neospora* is functionally antiviral, uninfected human fibroblasts or cells infected with *Neospora* or *Toxoplasma* were challenged with GFP-tagged Vesicular Stomatitis Virus (VSV), a standard virus used in bioassays for type I interferon [28]. Imaging these cells demonstrated that while VSV grew normally in naïve and *Toxoplasma*-infected cells, the virus was dramatically restricted in *Neospora*-infected cultures (Fig. 1d).

Viruses are known to infect various parasitic protozoa [29], in some cases influencing host transcriptional responses and promoting disease [30]. Several arguments indicate thatindicate t a cryptic *Neospora* virus is unlikely to be the source of the type I interferon response noted above. Analysis of raw trace reads from the *Toxoplasma* and *Neospora* genome projects [18] failed to identify evidence of viral DNA. Similarly, deep sequencing of both polyadenylated mRNA and small non-coding RNA libraries prepared from *Neospora* and *Toxoplasma* infected human cells yielded no significant evidence of viral RNA, based on mapping to NCBI RefSeq virus genomes and comparison with uninfected host cells (data not shown). Finally, total RNA isolated from *Toxoplasma* or *Neospora* and treated with S1 nuclease to degrade single stranded nucleic acid showed no evidence of double stranded RNA species smaller than 12 kb after staining with ethidium bromide (Fig. S1a). In both parasite species, S1 nuclease-resistant bands greater than 12kb (Fig. S1a) were DNase sensitive (Fig. S1b). In contrast, this approach readily detected double stranded RNA from the endogenous RNA virus, LRV1, present in *Leishmania guyanensis* (Fig. S1, arrows) [31]. Taken together, these data suggest that a parasite molecule present in *Neospora*, rather than an endogenous virus, is responsible for induction of a type I interferon response.

### *Toxoplasma* actively suppresses host cell induction of a type I interferon response

**Figure 2:**
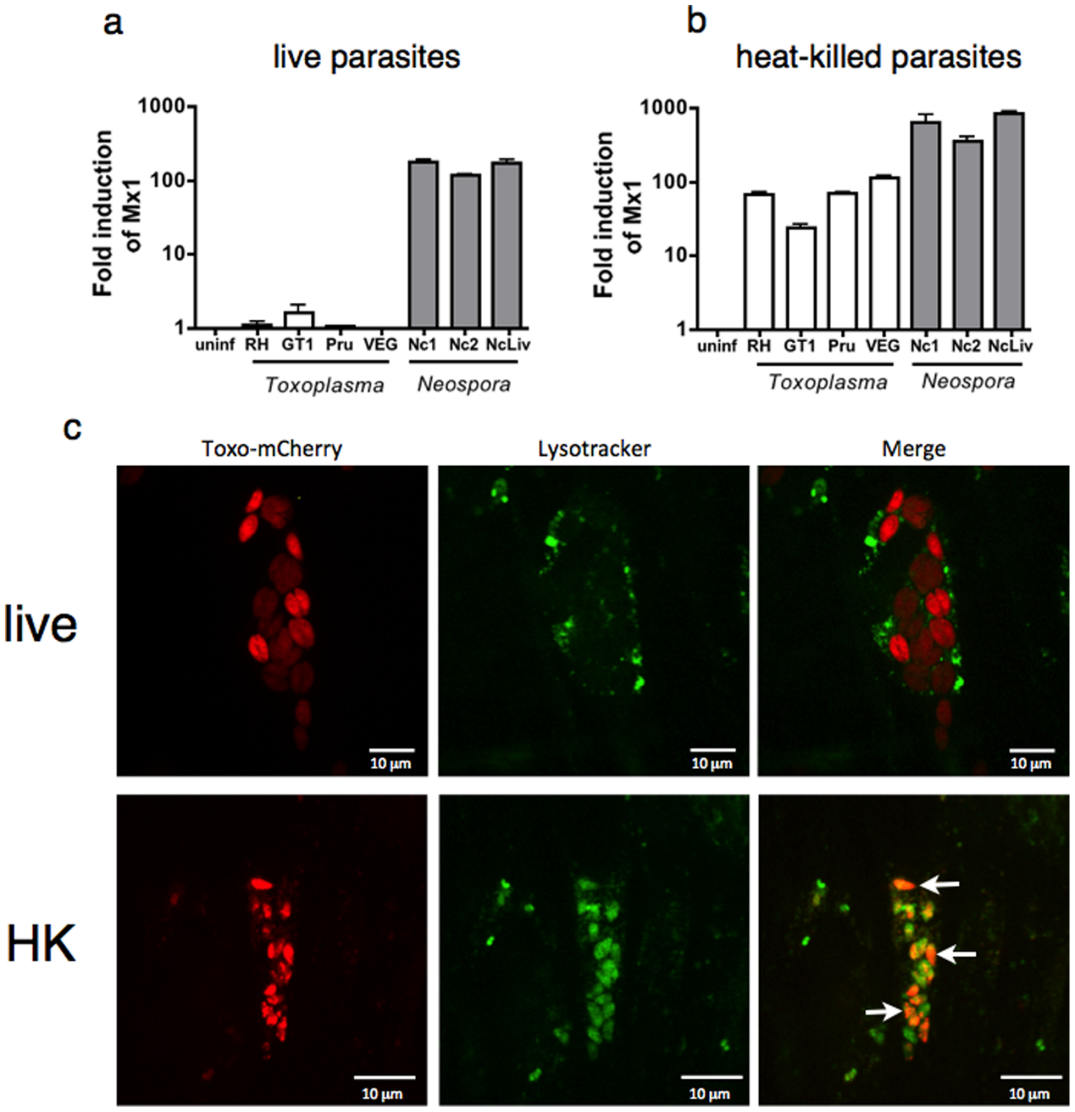
Active invasion is not required for innate recognition of parasites. QPCR analysis of the expression of the antiviral gene *Mx1* following (a) infection of HFF cells or (b) treatment of cells with heat-killed strains of *Toxoplasma* (open bars) or *Neospora* (shaded bars). (c) confocal fluorescence microscopy of HFF cells treated with live (top row) or heat killed (bottom row) *Toxoplasma*-mCherry. Lysosomes are stained with LysoTracker dye. Representative images are shown. Error bars indicate standard deviations for three biological replicates; * = *P*≤0.01. Experiments were repeated three times with similar results.

Using *Mx1* transcript abundance as a read-out for host response to parasite infection, three strains of *Neospora* and four strains of *Toxoplasma* were tested for induction of the type I interferon program. All *Neospora* isolates were potent inducers of *Mx1* expression, while no strain of *Toxoplasma* tested induced this response (Fig. 2a). Induction of *Mx1* correlated with parasite inoculum (Fig. S2a), suggesting that infected cells (rather than uninfected bystanders) are the likely the source of this response. Because *N. caninum* is a common pathogen of cattle rather than humans [20], [21], primary bovine fibroblasts were also infected with *Neospora,* demonstrating that both human and bovine cells upregulated *Mx1* expression following infection (Fig. S2b).

Many responses to *Toxoplasma* infection require active invasion of the host cell, including activation of the transcription factors STAT3 and EGR2, and suppression of proinflammatory signaling [32]–[34], indicating that these phenotypes are mediated by parasite factors secreted at the time of invasion or during intracellular replication. To test whether induction of the type I interferon profile also requires active infection, parasites were heat-killed prior to inoculation. Heat-killed *Neospora* retained their ability induce an antiviral program, even exhibiting enhanced *Mx1* induction compared to live parasites (Fig. 2b). Surprisingly, heat-killed *Toxoplasma* strains also induced *Mx1* expression, albeit at a level that averaged 11-fold weaker induction than seen with heat-killed *Neospora* strains. These data demonstrate that although both parasite species harbor a factor able to trigger the type I interferon response, *Neospora* elicits a much more robust response.

Given that innate induction of type I interferon is usually triggered by Toll-like receptors or RIG-I-like receptors (RIG-I and MDA5) that are located in the endo-lysosomal pathway or free in the cytosol, respectively, we were surprised that killed parasites not only induced a type I interferon profile, but did so more strongly than live parasites. We hypothesized that heat-killed parasites must get internalized by fibroblasts in order to access innate receptors that elicit this response. To test this idea, we used confocal fluorescence microscopy to visualize heat-killed *Toxoplasma* expressing mCherry in cells labeled with LysoTracker to identify lysosomes (Fig. 2c). While live *Toxoplasma* could be seen replicating in LysoTracker-negative vacuoles 18 hours after infection, heat-killed parasites were internalized and localized to LysoTracker positive compartments in the cell (Fig. 2c, arrows).

**Figure 3:**
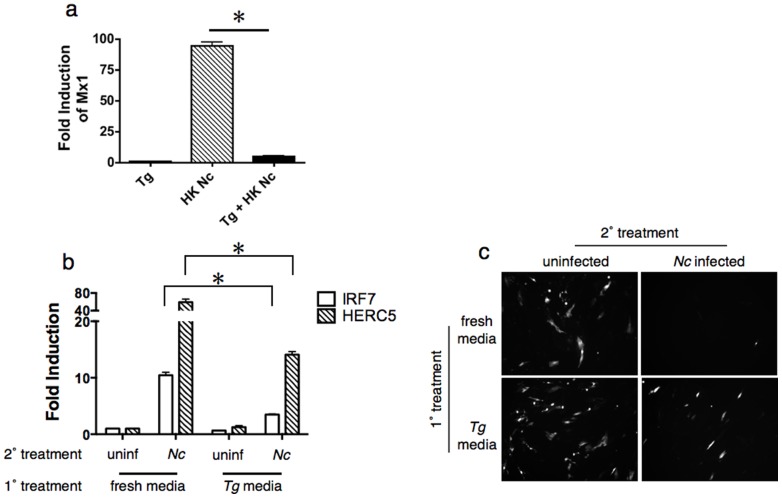
Toxoplasma actively suppresses anti-viral host responses via a soluble factor. (a) co-infection assay in HFF cells showing *Mx1* gene expression in *Toxoplasma* infected cultures (Tg), cultures inoculated with heat-killed *Neospora* (HK Nc; striped bar), or infected with *Toxoplasma* for one hr before challenge with heat-killed *Neospora* (black bar). (b) QPCR analysis of *IRF7* (open bars) and *HERC5* (striped bars) expression in HFF cells pre-treated with fresh media or conditioned media from *T. gondii* infected cells (1° treatment) prior to infection with *N. caninum* (2° treatment). c) HFF cells were pretreated with supernatants from experiment in panel a, then infected with VSV-GFP. Representative fluorescence micrographs are shown. Error bars indicate standard deviations for three biological replicates; * = *P*≤0.01. Experiment was repeated two times with similar results.

The observation that heat-killed, but not live, *Toxoplasma* induce *Mx1* expression suggested that live *T. gondii* actively suppressed this response. To test this hypothesis, human fibroblasts were first infected with live *Toxoplasma* (which does not induce *Mx1* expression), and then challenged 2 hr later with heat-killed *Neospora* (which do induce *Mx1*). Supernatants were collected at 24 hr after challenge, filtered to remove parasites, and transferred to naive fibroblast cultures to assay for type I interferon (Fig. 3a). While media from *Neospora* treated cultures was a potent inducer of *Mx1* expression, media from cells infected with *Toxoplasma* prior to *Neospora* exposure failed to elicit *Mx1* expression (Fig. 3a). These results demonstrate that *Toxoplasma* infection suppresses innate immune induction of type I interferon.

To test whether the suppressive effect observed in co-infection experiments was a cell-autonomous phenotype evident only in cells infected with *Toxoplasma*, conditioned media was recovered from *Toxoplasma* infected human fibroblasts, filtered to remove parasites, and used to pretreat naïve fibroblasts (Fig. 3b). Pretreatment of cells did not prevent subsequent infection with *Neospora* (not shown), but blocked *Neospora* induction of the antiviral genes *IRF7* and *HERC5*, 3-fold and 4-fold, respectively (Fig. 3b). Supernatants were filtered again and transferred to new cells to test for ability to restrict a viral challenge. As expected, cultures treated with supernatants from *Neospora* infected cells were completely refractory to a challenge with VSV-GFP (Fig. 3c, upper right). In contrast, supernatants recovered from cultures that were pretreated with *Toxoplasma* conditioned media prior to *Neospora* infection showed impaired virus restriction (Fig. 3c, lower right). Taken together, these data show that *Toxoplasma* infected cells produce a soluble factor that suppresses the innate induction of type I interferon.

### The macrophage response to Neospora infection is dependent on signaling through the type I interferon receptor

**Figure 4:**
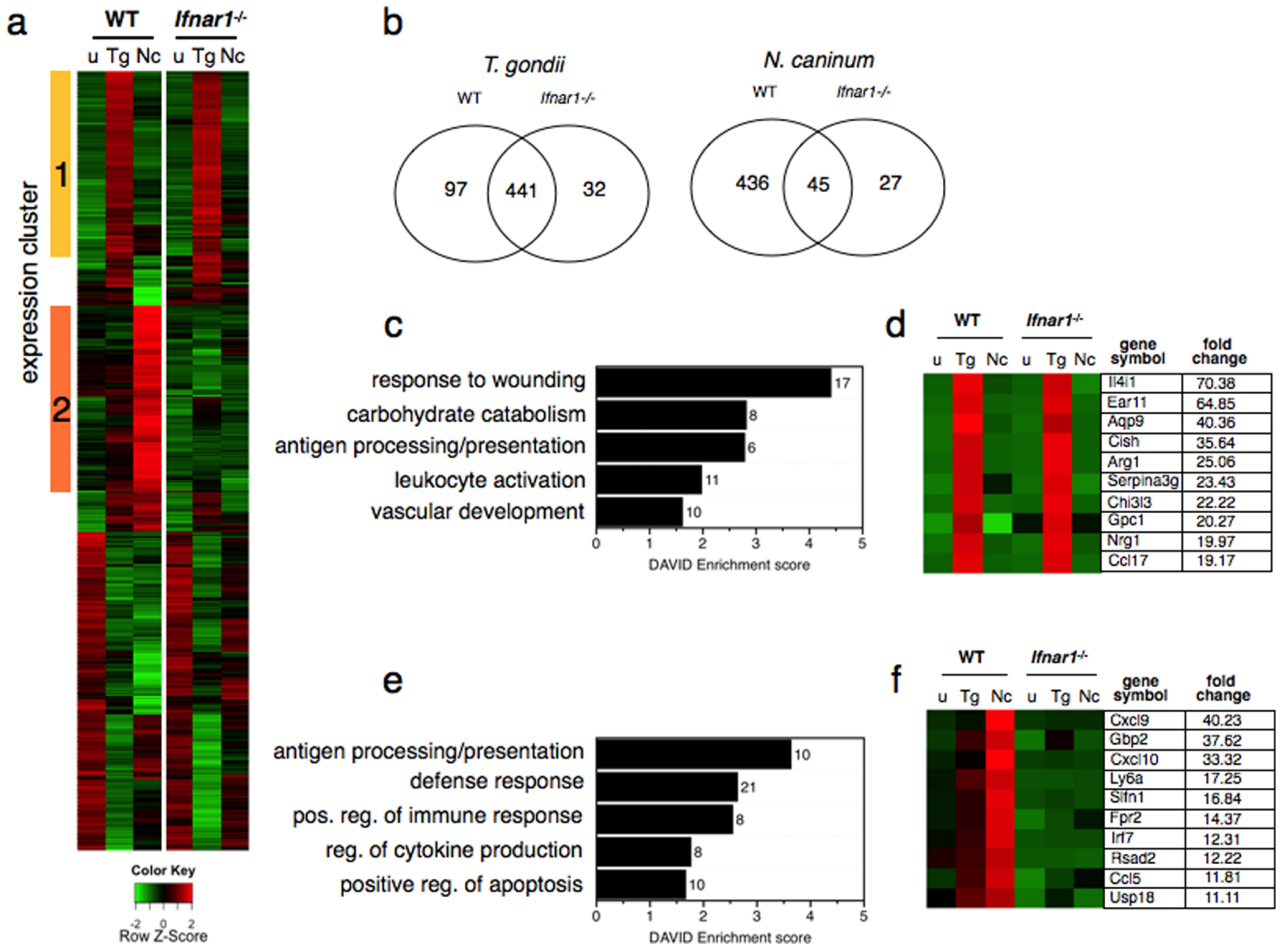
Macrophage transcriptional responses to Neospora infection are Ifnar1-dependent. Microarray-based expression profiling of *Neospora* (*Nc*) or *Toxoplasma* (*Tg*) infected wild-type or *Ifnar1-/-* bone marrow-derived mouse macrophages. a) heat map shows hierarchical clustering analysis of 833 genes differentially regulated by either Nc or Tg on either cell type relative to uninfected cells by ≥2-fold (FDR≤5%). Two clusters of co-regulated genes are indicated. Each column in heatmap represents an average of duplicate arrays, and color pattern represents row Z-score. b) Venn diagrams showing number of differentially regulated genes for each condition. c and e) GO enrichment results for cluster 1 and 2, respectively. d and f) heatmaps showing top ten most strongly induced genes in cluster 1 and 2, respectively. Gene symbol and fold change of infected WT relative to uninfected WT control are shown for each gene.

In order to determine the extent to which host transcriptional responses to *Neospora* and *Toxoplasma* (Fig. 1) are driven by type I interferon, bone marrow-derived murine macrophages were generated from either wild-type mice or mice lacking the type I interferon receptor subunit *Ifnar1* [35]. Transcriptional profiling of wild-type (WT) and *Ifnar1*-deficient macrophages infected with *Neospora* or *Toxoplasma* identified 833 genes differentially regulated (≥2-fold, FDR≤5%) compared to uninfected cells (Fig. 4a, Table S2). Although *Toxoplasma* elicited a similar host response on both cell types, the host response to *Neospora* was severely abrogated by the loss of type I interferon signaling (Fig. 4b), demonstrating that signaling through this receptor constitutes the major pathway responsible for shaping the transcriptional response to *N. caninum*.

Hierarchical clustering identified two distinct clusters of co-regulated genes (Fig. 4a). Cluster 1 (212 genes) was induced by *Toxoplasma*, but not *Neospora*, and was *Ifnar1*-independent. Gene ontology analysis showed that this cluster was strongly enriched for terms relating to wound healing (Fig. 4c), and arginase-1, Chi3l3, Ear11 and Il4i1 were amongst the most strongly induced genes in this cluster (Fig. 4d) - all well-known markers of alternative macrophage activation, which have recently been shown to be induced during infection with certain strains of *Toxoplasma* [36]. In contrast, cluster 2 (199 genes) was strongly induced by *Neospora*, yet only weakly induced by *Toxoplasma*, was *Ifnar1*-dependent (Fig. 4a), and was enriched for GO terms related to immune cell function and inflammation (Fig. 4e). The top ten most strongly induced genes in this cluster included key STAT1-dependent genes such as *Cxcl9* and *Cxcl10* as well as *Irf7*, a master regulator of antiviral gene transcription (Fig. 4f). Taken together with our data on human fibroblasts and bovine fibroblasts, these results show that *Neospora* induces a strong type I interferon signature in cells from diverse host species as well as immune and non-immune cell types, suggesting a conserved innate response to these parasites.

### The type I interferon response to Neospora is mediated by signaling through toll-like receptor 3 (TLR3) and the adaptor protein TRIF

**Figure 5:**
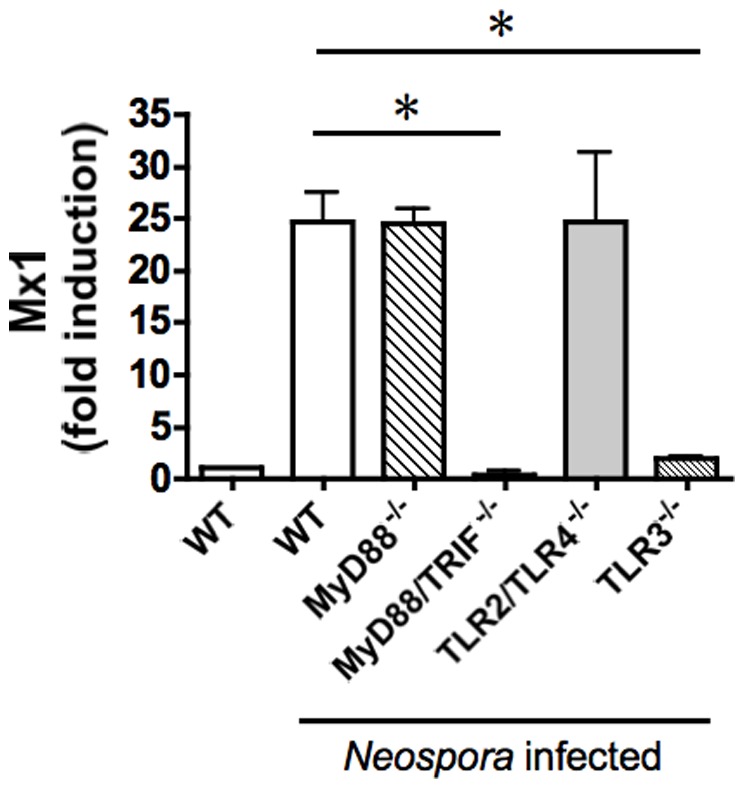
The Type I interferon response to Neospora infection is dependent on *Tlr3* and *Trif.* QPCR analysis of *Mx1* expression in *Neospora* infected bone marrow macrophages derived from wild-type (WT), *Myd88^−/−^*, *Myd88^−/−^/Trif^−/−^, Tlr2^−/−^/Tlr4^−/−^* and *Tlr3^−/−^* mice. Error bars indicate standard deviations for three biological replicates; * = *P*≤0.01. Experiment was repeated three times with similar results.

To identify the host pathway responsible for parasite-mediated induction of type I interferon, and downstream responses to this cytokine, bone marrow-derived macrophages were prepared from mice genetically deficient in various toll-like receptors or their adaptor proteins (Fig. 5). *Neospora* infection of *Myd88* deficient cells still resulted in induction of *Mx1* expression, ruling out a role for TLR1, 2, 5, 6, 7, 8, and 9 [37]. TLR4 is capable of inducing type I interferon production via either MYD88 or the alternative adapter TRIF [38], but infection of *Tlr2/Tlr4* double knock-out cells induced antiviral gene expression, ruling out a role for TLR4 as well. In contrast, cells lacking *Tlr3*, or lacking both *Myd88* and *Trif*, showed no induction of *Mx1* by live *Neospora. Tlr3^−/−^* and *Myd88^−/−^Trif^−/−^* cells also failed to respond to heat-killed parasites (not shown), demonstrating that both live and dead parasites are sensed through this pathway. Since TRIF is required for TLR3 signaling [39], [40], these data identify TLR3 - known to recognize double stranded RNA [41] - as the host receptor responsible for macrophage sensing of *Neospora* and type I interferon responses.

**Figure 6:**
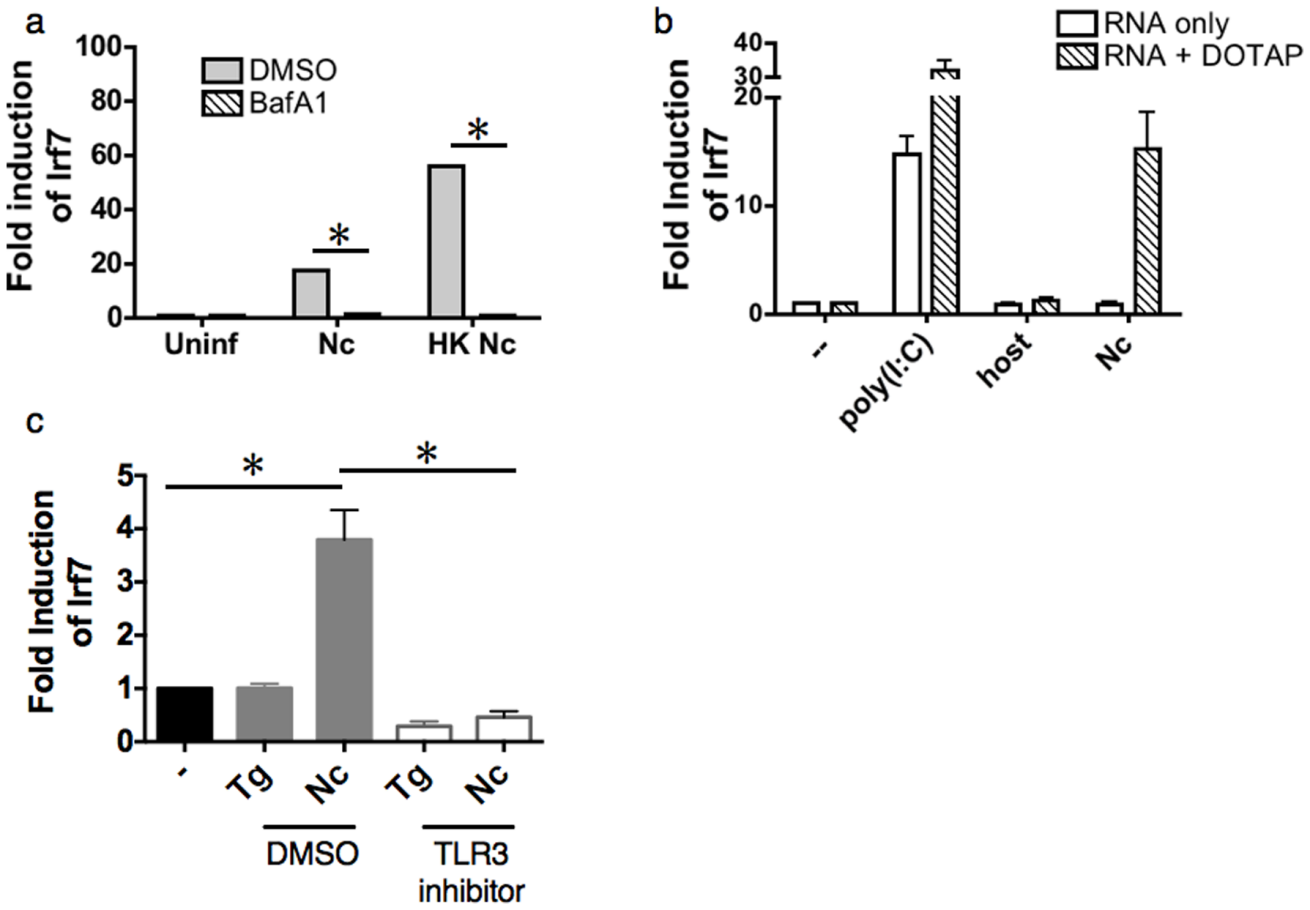
*Neospora* RNA elicits a TLR3-dependent type I interferon response. a) QPCR analysis of *Irf7* gene expression in wild-type mouse bone marrow-derived macrophages pretreated for 1 hr with either DMSO (control) or 100 nM bafilomycin A1 prior to 24 hr infection with live (Nc) or heat-killed *Neospora* (HK Nc). b) *Irf7* expression in *Myd88^−/−^* macrophages incubated with 1 µg/ml of either poly(I:C) or total RNA from HFF cells (host), or *Neospora* parasites, either alone (white) or in complex with DOTAP transfection reagent (striped), to enhance targeting of RNA to endosomes. c) *Irf7* expression in *Myd88^−/−^* macrophages incubated with 1 µg/ml of either *Toxoplasma* or *Neospora* RNA with DOTAP in cells pretreated with either DMSO or a small molecule inhibitor of TLR3/dsRNA complex formation. Error bars indicate standard deviations for three biological replicates; * = *P*≤0.01. Experiments were repeated two or three times with similar results.

Although TLR3 is primarily localized within endosomes, some cell types also express this receptor on the plasma membrane [42], [43]. To determine whether surface or endosomal TLR3 is responsible for sensing live and heat-killed *Neospora*, macrophages were pretreated with bafilomycin A1 (Fig. 6), a specific inhibitor of vacuolar ATPase that blocks endosomal acidification and subsequent fusion [44]. Although both live and heat-killed parasites induced the expression of *Mx1* and *Irf7* in macrophages treated with DMSO alone (Fig. 6a), this response was abolished in bafilomycin-treated cells, suggesting that endosomal TLR3 is responsible for recognizing both live and dead parasites in macrophages.

Since RNA is the prototypical ligand for TLR3 [41], parasite RNA was tested for its ability to activate this pathway. RNA extracted from *Neospora* or uninfected human cells (to control for any contamination of parasite material with host cell RNA) was added directly to *Myd88^−/−^* macrophage cultures (to exclude activation of TLR 7, 8 and 9, which also bind nucleic acids). None of the RNA preparations induced *Mx1* or *Irf7* expression, although the synthetic TLR3 ligand poly(I:C) resulted in 15-fold induction (Fig. 6b; white bars). Because it is unclear how efficiently extracellular RNA is taken up into the endosomes where TLR3 resides, parasite RNA was also complexed with the liposomal transfection reagent 1,2-dioleoyl-3-trimethylammonium-propane (DOTAP). When complexed with DOTAP, RNA from *Neospora*, but not *Toxoplasma*, strongly induced both *Irf7* (Fig. 6b and 6c) and *Mx1* (not shown). Thus, it appears that naked *Neospora* RNA is capable of triggering *Myd88*-independent type I interferon responses in macrophages, while RNA from *Toxoplasma* is not. Although the use of *Myd88^−/−^* cells in these experiments helped to narrow the range of TLRs that could be acting as receptors for *Neospora* RNA, it does not rule out a role for cytosolic RNA sensors. Therefore, we tested specifically whether TLR3 was the receptor for transfected parasite RNA by pre-incubating *Myd88^−/−^* macrophages with a small molecule competitive inhibitor of dsRNA binding to TLR3 [45]. In the presence of inhibitor, IRF7 induction by *Neospora* RNA was completely abolished, demonstrating that this is a TLR3-dependent process (Fig. 6c).

## Discussion

Despite the importance of protozoan parasites as causes of human and animal disease, relatively little information is available on innate signaling pathways triggered by these organisms, in contrast to the extensive literature on innate responses to viral and bacterial pathogens [14]. Glycosylphosphatidylinositol (GPI)-anchored surface antigens (found in many parasite species) can bind to TLR2 and/or TLR4 [46]; *Plasmodium* hemozoin can activate the inflammasome [47] and (when bound to DNA) TLR9 [48], [49]; and *Toxoplasma* profilin binds to TLR11 [13] and TLR12 [50], [51]. These interactions, in turn, trigger the production of pro-inflammatory cytokines, including IL-1, IL-6, TNF-α and IL-12. To this list, we can now add the induction of type I interferon responses (Fig. 1 and Fig. 4) via TLR3 and TRIF (Fig. 5), and our *in vitro* data suggest that parasite RNA is a potential trigger for this response (Fig. 6).

Infection by protozoan parasites has typically been associated with induction of type II (γ) interferon, while type I (α/β) interferon is prominent in viral infection, but type I responses to protozoa are not completely unprecedented. *Toxoplasma* infection elicits type I interferon from plasmacytoid dendritic cells [52], a cell type known to be a common source of this cytokine [53], while splenic red pulp macrophages produce type I interferon when exposed to *Plasmodium*-infected erythrocytes [54]-[56]. The parasite ligand was not identified in these studies, but the response was found to be *Tlr11*-dependent (*Toxoplasma*) and *Tlr9*-/*Myd88*-dependent (*Plasmodium*). More recently, a type I interferon transcriptional signature has been noted in hepatocytes infected with live (but not dead) *Plasmodium* sporozoites, involving the cytosolic RNA sensor MDA5 and the MAVS adaptor protein [57]. Although clearly distinct from the *Tlr3*- and *Trif*-dependent response to *Neospora* (and heat-killed *Toxoplasma*) reported above, these observations support the concept that protozoan parasites can be robust activators of type I interferon signaling (Fig. 4), and that mechanisms of innate sensing may vary depending on tissue or target cell type, parasite developmental stage, or other factors.

Although we found that both parasite species are capable of activating type I interferon responses when killed and added to cells directly, the level of induction was a log order of magnitude higher with *Neospora* (Fig. 2b). This may explain why transfection of macrophages with *Neospora* RNA, but not *Toxoplasma* RNA, was sufficient to elicit a TLR3-dependent induction of type I interferon responsive genes. These findings suggest that there are quantitative and/or qualitative differences in TLR3 ligand present in RNA from these parasite species. Both *Toxoplasma* and *Neospora* establish an intracellular ‘parasitophorous vacuole’ (PV) distinct from the endo-lysosomal pathway at the time of invasion [58], [59], raising the question of how parasite antigens, including nucleic acids, gain access to endosomal TLR3 or other intracellular pattern recognition receptors. One possibility is that TLR3 activation may be attributable to the release of RNA from parasites that are phagocytosed [60] or that die after invasion, rather than those engaged in productive intracellular replication. Alternatively, since the PV is intimately associated with the host mitochondria and endoplasmic reticulum [61], it is possible that RNA within the PV may access the host endo-lysosomal system (Fig. 6). PV antigens are known to enter the host endoplasmic reticulum for processing and presentation to T cells on MHC class I [62]–[64]. Endosomal vesicles may also be recruited to the PV [65], [66], facilitating the acquisition of nutrients, and perhaps playing a role in escape from the host cell [67], [68].

While type I and II interferons utilize different receptors and transcription factors, there is significant overlap in the genes that they induce [54], [69], suggesting some degree of functional redundancy. We observed that *Neospora* infection drives *Ifnar*-dependent expression of genes traditionally considered to be *Ifngr*-dependent (Fig. 4), including the chemokines *Cxcl9* and *Cxcl10*, and the GTPases *Igtp* and *Gbp2* (Fig. 4f), all of which have been shown to be important mediators of resistance to *Toxoplasma* infection [70]–[73]. Interestingly, Kim *et al*., found that *Ifnar^−/−^* deficiency only impacted *Plasmodium* infection when mice were also lacking the receptor for interferon-γ [54], consistent with the notion that the strong IFN-γ response typically seen in protozoan infections may mask a role for type I interferons. *In vivo* depletions of IFN-γ were carried out to test whether IFN-γ could compensate for loss of IFN-α signaling in *Neospora*-infected *Ifnar^−/−^* mice (Fig. S3). Although WT mice treated with control IgG had no parasites and limited neutrophilia evident in cytospins prepared from peritoneal exudate cells at 7 days post-infection (Fig. S3a), WT mice depleted of IFN-γ had abundant parasites and high neutrophil numbers evident (Fig. S3b), and acute mortality occurred at 8 dpi (data not shown). Parasites were not observed in *Ifnar^−/−^* mice treated with control IgG (Fig. S3c), however like their WT counterparts, all *Ifnar^−/−^* mice showed high parasitemia, high neutrophil infiltration and also succumbed to infection at 8 days post-infection (Fig. S3d). Taken together, these results are consistent with previous reports that IFN-γ is essential for control of *Neospora* [3], [74], and extend these findings by showing that type I interferon signaling is dispensable for the control of acute high-dose *Neospora* infection. Similar results have emerged for *Plasmodium* infection, where parasite burden and disease pathogenesis are either unchanged or minimally affected by loss of type I interferon signaling [54], [55].

The observation that live *Toxoplasma* potently suppresses the type I interferon pathway, and that this suppression is a dominant phenotype in co-infection assays with *Neospora* (Fig. 2) suggests at least two possible evolutionary scenarios: 1) that the common ancestor of these parasites could suppress induction of type I interferon and *Neospora* lost this ability after divergence; 2) or that the common ancestor could not suppress and *Toxoplasma* gained this ability after divergence. Such distinct host phenotypes could provide the basis for genetic screens or comparative genomic studies to identify the parasite factor(s) responsible for modulating the pathway. The fact that suppression is mediated by a soluble factor in the supernatant of *Toxoplasma* infected cells (Fig. 3) also raises the possibility that standard biochemical approaches could be used to identify and characterize a novel host or parasite factor that modifies this important signaling pathway. Our observation that *Toxoplasma* suppresses TLR3-dependent type I interferon induction *in vitro* contrasts with recent findings by Koblansky et al. [51] in which *Toxoplasma* profilin elicited low levels of IFN-α from dendritic cells in a *Tlr12*-dependent manner. Given that TLR12 signaling is MYD88-dependent, while TLR3 signaling in our model is not (Fig. 5), it stands to reason that *Toxoplasma* suppression may exhibit pathway specificity. In addition, Koblansky *et al*. attribute IFN-α production to plasmacytoid dendritic cells, and we did not test the induction or suppression of TLR3-dependent responses by either *Toxoplasma* or *Neospora* in this cell type. Interestingly, even low levels of IFN-α induced during Toxoplasma infection were critical in driving IFN-γ production by NK cells to limit parasite replication *in vivo* [51], providing a possible evolutionary pressure for *Toxoplasma* parasites to limit type I interferon induction. Taken together with our data, a broader picture of innate recognition of coccidian parasites begins to emerge in which both protein and nucleic acid ligands are capable of being recognized by distinct receptors, across various cell types and host species. A more complete understanding of how these pathogens are recognized by, and interfere with, innate signaling pathways will help to shed light on important aspects of the host-pathogen relationship during infections with protozoa.

## Materials and Methods

### Ethics statement

This study was carried out in accordance with the recommendations in the Guide for the Care and Use of Laboratory Animals of the National Institutes of Health. Protocols were approved by the Institutional Animal Care and Use (IACUC) committee of the University of Pennsylvania (animal welfare assurance number A3079-01). The University of Pennsylvania Animal Care and Use Programs are fully accredited by the Association for Assessment and Accreditation of Laboratory Animal Care International (AAALAC).

### Parasites, cells and mice

RH, GT1, Prugniaud (Pru), VEG strain T. gondii; and Nc1 [75], Nc2 [76] and NcLiv strain N. caninum were maintained by serial passage in human foreskin fibroblast (HFF) monolayers as described previously [77]. RH stably expressing mCherry were obtained from Dr. Anita Koshy [78]. Primary bovine fibroblasts were provided by Dr. Kenneth John McLaughlin (Penn). Cryopreserved bone marrow cells recovered from Myd88^−/−^, Myd88/Trif^−/−^, and Tlr2/Tlr4^−/−^ mice were differentiated into macrophages as described previously [79], and infected with a 1∶1 MOI of parasites for QPCR and microarray experiments at 16–22 hours post-infection. Ifnar1^−/−^ and Tlr3^−/−^ mice were provided by Drs. Hao Shen and Yongwon Choi (Penn), respectively. For mouse infections, WT and Ifnar1^−/−^ mice received either control IgG or anti-IFN-γ (clone XMG-1.2, 1mg I.P., BioXCell) on day 0 and again at 4 days post-infection. Mice were infected with 3×10^6^ NcLiv strain Neospora caninum by I.P. injection on day 0. All mice were maintained at the University of Pennsylvania in accordance with Institutional Animal Care and Use Committee guidelines. For cytospins, 200,000 cells were spun onto glass slides using a Shandon Cytospin and stained with Diff-Quik, dehydrated, mounted with Permount (Sigma Aldrich), and images captured on a Nikon E600 upright microscope.

### Microarray-based expression profiling and analysis of Gene Ontology enrichment

For whole genome expression microarray, total RNA was isolated using the RNeasy Plus kit (Qiagen). Biotin-labeled complementary RNA (cRNA) was generated using the Illumina TotalPrep RNA amplification kit. RNA and cRNA quality were assessed on a BioAnalyzer (Agilent). Illumina HumanRef-8 version 3 (for HFF cells) or MouseWG-6 version 2 (for bone marrow macrophages) expression beadchips were hybridized with cRNA from two to three biological replicates per condition according to the manufacturer′s instructions, and scanned on a beadscan unit. Scanned images were converted to raw expression values using GenomeStudio v1.8 software (Illumina). Data analysis was carried out using the statistical computing environment, R (v3.0.2), the Bioconductor suite of packages for R, and RStudio (v0.97). Raw data was background subtracted, variance stabilized and normalized by robust spline normalization using the Lumi package [80]. Differentially expressed genes were identified by linear modeling and Bayesian statistics using the Limma package [81], [82]. Probes sets that were differentially regulated >2fold (FDR<5%; after controlling for multiple testing using the Benjamini-Hochberg method [83], [84]) were used for hierarchical clustering and heatmap generation in R. Multiple probe sets per gene were collapsed to a single non-redundant list of expression data using the “MaxMean” method of the CollapseRows function in R [85]. For each gene, a mean expression value was calculated for biological replicates and used for heatmap generation (Fig. 2 and Fig. 4). Clusters of co-regulated genes were identified by pearson correlation using the hclust function of the stats package in R. Data has been deposited on the Gene Expression Omnibus (GEO) database for public access (GSE45632 and GSE45633). Gene Ontology enrichment analysis was conducted using either the Function Annotation Chart (Fig. 1c) or Functional Annotation Clustering (Fig. 4) tools using only GO BP ‘fat’ terms in the Database for Visualization and Integrative Discovery (DAVID) [86], [87]. DAVID enrichment scores >1.3 are equivalent to a P value<0.05 (Fig. 4c and 4e).

### Quantitative PCR (QPCR)

cDNA was generated from total RNA using the High Capacity cDNA Reverse Transcription Kit (Applied Biosystems). QPCR was carried out using SYBR green dye and gene specific primers for human or mouse MX1 and IRF7. Relative transcript abundance was determined using the ΔΔCt method [88], using a standard curve of cDNA amplified with primers specific for the house-keeping gene GAPDH.

### Viral interference assays

For Fig. 1d, confluent HFF cells were infected with *Toxoplasma* or *Neospora* for 16 hr, followed by challenge with GFP-tagged vesicular stomatitis virus (VSV; MOI = 20 pfu/host cell) for 8 hr prior to assaying by fluorescence microscopy. For Fig. 3, HFF cells were pretreated for 4 hours with either fresh media or with conditioned media recovered from *Toxoplasma* infected HFF cells at 24 hr post-infection and filtered to remove parasites and host cell debris. Cells were then infected with *Neospora* for 24 hours and induction of *IRF7* and *HERC5* transcript levels was measured by QPCR (Fig. 3a). Supernatants from these cultures were also recovered, filtered again through a 0.22 µm filter to remove parasites and host cell debris, and transferred to fresh HFF cell monolayers prior to VSV-GFP challenge (Fig. 3b).

### Bafilomycin treatment, DOTAP transfections and TLR3/dsRNA antagonist

Wild-type bone marrow-derived macrophages were treated with 100 nM Bafilomycin A1 for 1 hr at 37°C to block endosomal fusion prior to infection, RNA harvest and QPCR as described above. For transfection of macrophages with RNA, parasite or host (HFF) total RNA was isolated using the miRNeasy kit (Qiagen). *Myd88* ^−/−^ bone marrow-derived macrophages were used in order to minimize induction of innate signaling pathways other than *Tlr3*. 1 µg total RNA or poly(I:C) (Imgenex) was added to each chamber of a 24-well plate containing 1 ml of media and 1×10^6^ *Myd88* ^−/−^ macrophages. For more efficient targeting to endosomes, 1 µg of parasite RNA, host RNA, or poly(I:C) was mixed with 10 µg 1,2-dioleoyl-3-trimethylammonium-propane (DOTAP) liposomal transfection reagent (Roche Applied Science). Cells were incubated for 18 hr with transfection mixture and RNA was isolated for QPCR analysis as described above. To test a role for TLR3 in recognition of parasite RNA, a thiophenecarboxamidopropionate small molecule inhibitor of the TLR3/dsRNA complex (Calibochem) was added to cultures at 50 nM for 1hr at 37°C before transfection of cells with RNA/DOTAP.

### Assaying for presence of parasite RNA virus

To test for presence of an endogenous RNA virus in the parasite, total RNA was extracted from purified *Toxoplasma* and *Neospora* tachyzoites using Trizol. Nuclease digestions were carried out using 60 µg of total RNA in 15 µl of volume containing 3 units/µl of S1 nuclease (Invitrogen), alone or following Dnase treatment and RNA purification, and incubated at 37°C for 45 minutes. Samples were analyzed on 0.8% native agarose in 1× TAE buffer at 4°C. S1 nuclease-treated *Leishmania guyanensis* (strain M4147), which contains the endogenous RNA virus LRV1, was used as a positive control.

### Fluorescence microscopy to assess parasite internalization

HFF cells grown to confluency on coverslips were infected with a 3∶1 MOI of either live or heat-killed (56°C for 30 minutes) RH strain *Toxoplasma* expressing mCherry [78]. 18 hours after infection, coverslips incubated with 50 nM LysoTracker (Molecular Probes) for 10 minutes at 37°C were live mounted, and immediately imaged using a Leica DMI400 with Yokagawa spinning disk confocal system. Images were overlayed and analyzed using Image J software, v1.47.

### Statistical Analysis and data visualization

All experiments were repeated 2-4 times, and means and standard deviations were calculated from biological replicates. Significance was determined using a Student's t-test. Statistical analysis was carried out using Prism 4 (GraphPad Software) and data visualization using DataGraph 3.0 (Visual Data Tools).

## Supporting Information

Figure S1 **Assaying for double stranded RNA in** ***Toxoplasma*** **and** ***Neospora*** **.** 0.8% agarose gel electrophoresis and ethidium bromide staining of *Leishmania guyanensis* (*Lg*), *Toxoplasma gondii* (*Tg*) and *Neospora caninum* (*Nc*) total RNAs treated with A) S1 nuclease, B) DNase I and S1 nuclease, C) untreated total RNA of *Toxoplasma gondii* (*Tg*) and *Neospora caninum* (*Nc*). 28S and 18S rRNAs bands sensitive to S1 nuclease shown as loading control. DNA ladder (L) with kilobase (kb) markers is shown. Arrow indicates an S1 nuclease- and Dnase-resistant double stranded RNA from *L. guyanensis* that corresponds to a known endogenous RNA virus [31].
(TIFF)

Figure S2 **Host response to** ***Neospora*** **infection correlates with inoculum and is not restricted to human cells.** QPCR analysis of the expression of the antiviral gene *Mx1* in (a) HFF cells infected with *Neospora* tachyzoites at various multiplicity of infection (MOI), and (b) bovine primary fibroblasts infected with either *Toxoplasma* (Tg) or *Neospora* (Nc). Similar results were obtained for *Irf7* (not shown). Error bars indicate standard deviations for two biological replicates; * = *P*≤0.01.
(TIFF)

Figure S3 **IFN-γ, but not type I interferons, are essential for control of high-dose** ***Neospora*** **infection** ***in vivo*** **.** Diff-quik stained cytospins of peritoneal exudate cells recovered from (a-b) WT and (c-d) *Ifnar1* ^-/-^ mice (7 days post-infection) treated with either control IgG or neutralizing antibody to IFN-γ (clone XMG-1.2). Experiments were repeated two times, with 4-5 mice per group. Representative micrographs are shown. Arrows indicate parasite-infected cells.
(TIFF)

Table S1 Illumina microarray average log2 expression values from Figure 1a showing 822 human genes differentially regulated ≥2-fold (FDR≤5%) following infection of human foreskin fibroblast (HFF) cells with either *Neospora* or representative *Toxoplasma* strains. 66 *Neospora*-specific antiviral genes (Figure 1b) are shown on separate tab of this Excel file.
(XLSX)

Table S2 **Illumina microarray average log2 expression values from** **Figure 4a** **showing 833 mouse genes differentially regulated ≥2-fold (FDR≤5%) following infection of wild-type and** ***Ifnar1*** **^-/-^ bone marrow-derived macrophages with either** ***Neospora*** **(Nc2 strain) or** ***Toxoplasma*** **(VEG strain).** *Toxoplasma*-specific, *Ifnar1*-independent genes (Fig. 4a, cluster 1; 212 genes) and *Neospora* specific, *Ifnar1*-dependent genes (Fig. 4a, cluster 2; 199 genes) are shown on separate tabs of this Excel file.
(XLSX)

## References

[1] Kotloff KL, Nataro JP, Blackwelder WC, Nasrin D, Farag TH, et al (2013) Burden and aetiology of diarrhoeal disease in infants and young children in developing countries (the Global Enteric Multicenter Study, GEMS): a prospective, case-control study. Lancet 382: 209–222. 23680352 10.1016/S0140-6736(13)60844-2

[2] Mead PS, Slutsker L, Dietz V, McCaig LF, Bresee JS, et al (1999) Food-related illness and death in the United States. Emerg Infect Dis 5: 607–625. 10511517 10.3201/eid0505.990502 PMC2627714

[3] Suzuki Y, Orellana MA, Schreiber RD, Remington JS (1988) Interferon-gamma: the major mediator of resistance against Toxoplasma gondii. Science 240: 516–518. 3128869 10.1126/science.3128869

[4] Yap GS, Sher A (1999) Effector cells of both nonhemopoietic and hemopoietic origin are required for interferon (IFN)-gamma- and tumor necrosis factor (TNF)-alpha-dependent host resistance to the intracellular pathogen, Toxoplasma gondii. J Exp Med 189: 1083–1092. 10190899 10.1084/jem.189.7.1083 PMC2192999

[5] Hayward AR, Chmura K, Cosyns M (2000) Interferon-gamma is required for innate immunity to Cryptosporidium parvum in mice. J Infect Dis 182: 1001–1004. 10950807 10.1086/315802

[6] Wang ZE, Reiner SL, Zheng S, Dalton DK, Locksley RM (1994) CD4+ effector cells default to the Th2 pathway in interferon gamma-deficient mice infected with Leishmania major. J Exp Med 179: 1367–1371. 7908325 10.1084/jem.179.4.1367 PMC2191434

[7] Silva JS, Morrissey PJ, Grabstein KH, Mohler KM, Anderson D, et al (1992) Interleukin 10 and interferon gamma regulation of experimental Trypanosoma cruzi infection. J Exp Med 175: 169–174. 1730915 10.1084/jem.175.1.169 PMC2119081

[8] Hou B, Benson A, Kuzmich L, DeFranco AL, Yarovinsky F (2011) Critical coordination of innate immune defense against Toxoplasma gondii by dendritic cells responding via their Toll-like receptors. Proc Natl Acad Sci U S A 108: 278–283. 21173242 10.1073/pnas.1011549108 PMC3017180

[9] Denkers EY, Gazzinelli RT, Martin D, Sher A (1993) Emergence of NK1.1+ cells as effectors of IFN-gamma dependent immunity to Toxoplasma gondii in MHC class I-deficient mice. J Exp Med 178: 1465–1472. 8228800 10.1084/jem.178.5.1465 PMC2191244

[10] Gazzinelli RT, Wysocka M, Hayashi S, Denkers EY, Hieny S, et al (1994) Parasite-induced IL-12 stimulates early IFN-gamma synthesis and resistance during acute infection with Toxoplasma gondii. J Immunol 153: 2533–2543. 7915739

[11] Bliss SK, Butcher BA, Denkers EY (2000) Rapid recruitment of neutrophils containing prestored IL-12 during microbial infection. J Immunol 165: 4515–4521. 11035091 10.4049/jimmunol.165.8.4515

[12] Bliss SK, Zhang Y, Denkers EY (1999) Murine neutrophil stimulation by Toxoplasma gondii antigen drives high level production of IFN-gamma-independent IL-12. J Immunol 163: 2081–2088. 10438947

[13] Yarovinsky F, Zhang D, Andersen JF, Bannenberg GL, Serhan CN, et al (2005) TLR11 activation of dendritic cells by a protozoan profilin-like protein. Science 308: 1626–1629. 15860593 10.1126/science.1109893

[14] Lee CC, Avalos AM, Ploegh HL (2012) Accessory molecules for Toll-like receptors and their function. Nat Rev Immunol 12: 168–179. 22301850 10.1038/nri3151 PMC3677579

[15] Gazzinelli RT, Denkers EY (2006) Protozoan encounters with Toll-like receptor signalling pathways: implications for host parasitism. Nat Rev Immunol 6: 895–906. 17110955 10.1038/nri1978

[16] Dubey JP, Carpenter JL, Speer CA, Topper MJ, Uggla A (1988) Newly recognized fatal protozoan disease of dogs. J Am Vet Med Assoc 192: 1269–1285. 3391851

[17] Speer CA, Dubey JP, McAllister MM, Blixt JA (1999) Comparative ultrastructure of tachyzoites, bradyzoites, and tissue cysts of Neospora caninum and Toxoplasma gondii. Int J Parasitol 29: 1509–1519. 10608436 10.1016/s0020-7519(99)00132-0

[18] Reid AJ, Vermont SJ, Cotton JA, Harris D, Hill-Cawthorne GA, et al (2012) Comparative genomics of the apicomplexan parasites Toxoplasma gondii and Neospora caninum: Coccidia differing in host range and transmission strategy. PLoS Pathog 8: e1002567. 22457617 10.1371/journal.ppat.1002567 PMC3310773

[19] Anderson ML, Reynolds JP, Rowe JD, Sverlow KW, Packham AE, et al (1997) Evidence of vertical transmission of Neospora sp infection in dairy cattle. J Am Vet Med Assoc 210: 1169–1172. 9108925

[20] McCann CM, Vyse AJ, Salmon RL, Thomas D, Williams DJ, et al (2008) Lack of serologic evidence of Neospora caninum in humans, England. Emerg Infect Dis 14: 978–980. 18507920 10.3201/eid1406.071128 PMC2600293

[21] Robert-Gangneux F, Klein F (2009) Serologic screening for Neospora caninum, France. Emerg Infect Dis 15: 987–989. 19523316 10.3201/eid1506.081414 PMC2727314

[22] Howe DK, Sibley LD (1995) Toxoplasma gondii comprises three clonal lineages: correlation of parasite genotype with human disease. J Infect Dis 172: 1561–1566. 7594717 10.1093/infdis/172.6.1561

[23] Su C, Khan A, Zhou P, Majumdar D, Ajzenberg D, et al (2012) Globally diverse Toxoplasma gondii isolates comprise six major clades originating from a small number of distinct ancestral lineages. Proc Natl Acad Sci U S A 109: 5844–5849. 22431627 10.1073/pnas.1203190109 PMC3326454

[24] Ashburner M, Ball CA, Blake JA, Botstein D, Butler H, et al (2000) Gene ontology: tool for the unification of biology. The Gene Ontology Consortium. Nat Genet 25: 25–29. 10802651 10.1038/75556 PMC3037419

[25] Lindenmann J (1964) Inheritance of Resistance to Influenza Virus in Mice. Proc Soc Exp Biol Med 116: 506–509. 14193387 10.3181/00379727-116-29292

[26] Staeheli P, Haller O, Boll W, Lindenmann J, Weissmann C (1986) Mx protein: constitutive expression in 3T3 cells transformed with cloned Mx cDNA confers selective resistance to influenza virus. Cell 44: 147–158. 3000619 10.1016/0092-8674(86)90493-9

[27] Nakayama M, Nagata K, Kato A, Ishihama A (1991) Interferon-inducible mouse Mx1 protein that confers resistance to influenza virus is GTPase. J Biol Chem 266: 21404–21408. 1657964

[28] Meager A (2002) Biological assays for interferons. J Immunol Methods 261: 21–36. 11861063 10.1016/s0022-1759(01)00570-1

[29] Wang AL, Wang CC (1991) Viruses of the protozoa. Annu Rev Microbiol 45: 251–263. 1741616 10.1146/annurev.mi.45.100191.001343

[30] Ives A, Ronet C, Prevel F, Ruzzante G, Fuertes-Marraco S, et al (2011) Leishmania RNA virus controls the severity of mucocutaneous leishmaniasis. Science 331: 775–778. 21311023 10.1126/science.1199326 PMC3253482

[31] Zangger H, Ronet C, Desponds C, Kuhlmann FM, Robinson J, et al (2013) Detection of Leishmania RNA virus in Leishmania parasites. PLoS Negl Trop Dis 7: e2006. 23326619 10.1371/journal.pntd.0002006 PMC3542153

[32] Butcher BA, Denkers EY (2002) Mechanism of entry determines the ability of Toxoplasma gondii to inhibit macrophage proinflammatory cytokine production. Infect Immun 70: 5216–5224. 12183573 10.1128/IAI.70.9.5216-5224.2002 PMC128277

[33] Butcher BA, Kim L, Panopoulos AD, Watowich SS, Murray PJ, et al (2005) IL-10-independent STAT3 activation by Toxoplasma gondii mediates suppression of IL-12 and TNF-alpha in host macrophages. J Immunol 174: 3148–3152. 15749841 10.4049/jimmunol.174.6.3148

[34] Phelps ED, Sweeney KR, Blader IJ (2008) Toxoplasma gondii rhoptry discharge correlates with activation of the early growth response 2 host cell transcription factor. Infect Immun 76: 4703–4712. 18678671 10.1128/IAI.01447-07 PMC2546823

[35] Muller U, Steinhoff U, Reis LF, Hemmi S, Pavlovic J, et al (1994) Functional role of type I and type II interferons in antiviral defense. Science 264: 1918–1921. 8009221 10.1126/science.8009221

[36] Jensen KD, Wang Y, Wojno ED, Shastri AJ, Hu K, et al (2011) Toxoplasma polymorphic effectors determine macrophage polarization and intestinal inflammation. Cell Host Microbe 9: 472–483. 21669396 10.1016/j.chom.2011.04.015 PMC3131154

[37] O′Neill LA, Bowie AG (2007) The family of five: TIR-domain-containing adaptors in Toll-like receptor signalling. Nat Rev Immunol 7: 353–364. 17457343 10.1038/nri2079

[38] Yamamoto M, Sato S, Hemmi H, Hoshino K, Kaisho T, et al (2003) Role of adaptor TRIF in the MyD88-independent toll-like receptor signaling pathway. Science 301: 640–643. 12855817 10.1126/science.1087262

[39] Hoebe K, Du X, Georgel P, Janssen E, Tabeta K, et al (2003) Identification of Lps2 as a key transducer of MyD88-independent TIR signalling. Nature 424: 743–748. 12872135 10.1038/nature01889

[40] Yamamoto M, Sato S, Mori K, Hoshino K, Takeuchi O, et al (2002) Cutting edge: a novel Toll/IL-1 receptor domain-containing adapter that preferentially activates the IFN-beta promoter in the Toll-like receptor signaling. J Immunol 169: 6668–6672. 12471095 10.4049/jimmunol.169.12.6668

[41] Alexopoulou L, Holt AC, Medzhitov R, Flavell RA (2001) Recognition of double-stranded RNA and activation of NF-kappaB by Toll-like receptor 3. Nature 413: 732–738. 11607032 10.1038/35099560

[42] Orinska Z, Bulanova E, Budagian V, Metz M, Maurer M, et al (2005) TLR3-induced activation of mast cells modulates CD8+ T-cell recruitment. Blood 106: 978–987. 15840693 10.1182/blood-2004-07-2656

[43] Jack CS, Arbour N, Manusow J, Montgrain V, Blain M, et al (2005) TLR signaling tailors innate immune responses in human microglia and astrocytes. J Immunol 175: 4320–4330. 16177072 10.4049/jimmunol.175.7.4320

[44] Yamamoto A, Tagawa Y, Yoshimori T, Moriyama Y, Masaki R, et al (1998) Bafilomycin A1 prevents maturation of autophagic vacuoles by inhibiting fusion between autophagosomes and lysosomes in rat hepatoma cell line, H-4-II-E cells. Cell Struct Funct 23: 33–42. 9639028 10.1247/csf.23.33

[45] Cheng K, Wang X, Yin H (2011) Small-molecule inhibitors of the TLR3/dsRNA complex. J Am Chem Soc 133: 3764–3767. 21355588 10.1021/ja111312h PMC3068529

[46] Debierre-Grockiego F, Schwarz RT (2010) Immunological reactions in response to apicomplexan glycosylphosphatidylinositols. Glycobiology 20: 801–811. 20378610 10.1093/glycob/cwq038

[47] Griffith JW, Sun T, McIntosh MT, Bucala R (2009) Pure Hemozoin is inflammatory in vivo and activates the NALP3 inflammasome via release of uric acid. J Immunol 183: 5208–5220. 19783673 10.4049/jimmunol.0713552 PMC3612522

[48] Parroche P, Lauw FN, Goutagny N, Latz E, Monks BG, et al (2007) Malaria hemozoin is immunologically inert but radically enhances innate responses by presenting malaria DNA to Toll-like receptor 9. Proc Natl Acad Sci U S A 104: 1919–1924. 17261807 10.1073/pnas.0608745104 PMC1794278

[49] Coban C, Ishii KJ, Kawai T, Hemmi H, Sato S, et al (2005) Toll-like receptor 9 mediates innate immune activation by the malaria pigment hemozoin. J Exp Med 201: 19–25. 15630134 10.1084/jem.20041836 PMC2212757

[50] Andrade WA, Souza Mdo C, Ramos-Martinez E, Nagpal K, Dutra MS, et al (2013) Combined action of nucleic acid-sensing Toll-like receptors and TLR11/TLR12 heterodimers imparts resistance to Toxoplasma gondii in mice. Cell Host Microbe 13: 42–53. 23290966 10.1016/j.chom.2012.12.003 PMC3552114

[51] Koblansky AA, Jankovic D, Oh H, Hieny S, Sungnak W, et al (2013) Recognition of profilin by Toll-like receptor 12 is critical for host resistance to Toxoplasma gondii. Immunity 38: 119–130. 23246311 10.1016/j.immuni.2012.09.016 PMC3601573

[52] Pepper M, Dzierszinski F, Wilson E, Tait E, Fang Q, et al (2008) Plasmacytoid dendritic cells are activated by Toxoplasma gondii to present antigen and produce cytokines. J Immunol 180: 6229–6236. 18424745 10.4049/jimmunol.180.9.6229 PMC6157020

[53] Asselin-Paturel C, Boonstra A, Dalod M, Durand I, Yessaad N, et al (2001) Mouse type I IFN-producing cells are immature APCs with plasmacytoid morphology. Nat Immunol 2: 1144–1150. 11713464 10.1038/ni736

[54] Kim CC, Nelson CS, Wilson EB, Hou B, DeFranco AL, et al (2012) Splenic red pulp macrophages produce type I interferons as early sentinels of malaria infection but are dispensable for control. PLoS One 7: e48126. 23144737 10.1371/journal.pone.0048126 PMC3483282

[55] Voisine C, Mastelic B, Sponaas AM, Langhorne J (2010) Classical CD11c+ dendritic cells, not plasmacytoid dendritic cells, induce T cell responses to Plasmodium chabaudi malaria. Int J Parasitol 40: 711–719. 19968996 10.1016/j.ijpara.2009.11.005

[56] Pichyangkul S, Yongvanitchit K, Kum-arb U, Hemmi H, Akira S, et al (2004) Malaria blood stage parasites activate human plasmacytoid dendritic cells and murine dendritic cells through a Toll-like receptor 9-dependent pathway. J Immunol 172: 4926–4933. 15067072 10.4049/jimmunol.172.8.4926

[57] Liehl P, Zuzarte-Luis V, Chan J, Zillinger T, Baptista F, et al.. (2013) Host-cell sensors for Plasmodium activate innate immunity against liver-stage infection. Nat Med. 10.1038/nm.3424 PMC4096771 24362933

[58] Sibley LD, Weidner E, Krahenbuhl JL (1985) Phagosome acidification blocked by intracellular Toxoplasma gondii. Nature 315: 416–419. 2860567 10.1038/315416a0

[59] Joiner KA, Fuhrman SA, Miettinen HM, Kasper LH, Mellman I (1990) Toxoplasma gondii: fusion competence of parasitophorous vacuoles in Fc receptor-transfected fibroblasts. Science 249: 641–646. 2200126 10.1126/science.2200126

[60] Morisaki JH, Heuser JE, Sibley LD (1995) Invasion of Toxoplasma gondii occurs by active penetration of the host cell. J Cell Sci 108 (Pt 6): 2457–2464. 10.1242/jcs.108.6.2457 7673360

[61] Sinai AP, Webster P, Joiner KA (1997) Association of host cell endoplasmic reticulum and mitochondria with the Toxoplasma gondii parasitophorous vacuole membrane: a high affinity interaction. J Cell Sci 110 (Pt 17): 2117–2128. 10.1242/jcs.110.17.2117 9378762

[62] Goldszmid RS, Coppens I, Lev A, Caspar P, Mellman I, et al (2009) Host ER-parasitophorous vacuole interaction provides a route of entry for antigen cross-presentation in Toxoplasma gondii-infected dendritic cells. J Exp Med 206: 399–410. 19153244 10.1084/jem.20082108 PMC2646567

[63] Gubbels MJ, Striepen B, Shastri N, Turkoz M, Robey EA (2005) Class I major histocompatibility complex presentation of antigens that escape from the parasitophorous vacuole of Toxoplasma gondii. Infect Immun 73: 703–711. 15664908 10.1128/IAI.73.2.703-711.2005 PMC547086

[64] Dzierszinski F, Pepper M, Stumhofer JS, LaRosa DF, Wilson EH, et al (2007) Presentation of Toxoplasma gondii antigens via the endogenous major histocompatibility complex class I pathway in nonprofessional and professional antigen-presenting cells. Infect Immun 75: 5200–5209. 17846116 10.1128/IAI.00954-07 PMC2168266

[65] Mercier C, Dubremetz JF, Rauscher B, Lecordier L, Sibley LD, et al (2002) Biogenesis of nanotubular network in Toxoplasma parasitophorous vacuole induced by parasite proteins. Mol Biol Cell 13: 2397–2409. 12134078 10.1091/mbc.E02-01-0021 PMC117322

[66] Sibley LD, Niesman IR, Parmley SF, Cesbron-Delauw MF (1995) Regulated secretion of multi-lamellar vesicles leads to formation of a tubulo-vesicular network in host-cell vacuoles occupied by Toxoplasma gondii. J Cell Sci 108 (Pt 4): 1669–1677. 10.1242/jcs.108.4.1669 7615684

[67] Caffaro CE, Boothroyd JC (2011) Evidence for host cells as the major contributor of lipids in the intravacuolar network of Toxoplasma-infected cells. Eukaryot Cell 10: 1095–1099. 21685319 10.1128/EC.00002-11 PMC3165450

[68] Coppens I, Sinai AP, Joiner KA (2000) Toxoplasma gondii exploits host low-density lipoprotein receptor-mediated endocytosis for cholesterol acquisition. J Cell Biol 149: 167–180. 10747095 10.1083/jcb.149.1.167 PMC2175092

[69] Hertzog P, Forster S, Samarajiwa S (2011) Systems biology of interferon responses. J Interferon Cytokine Res 31: 5–11. 21226606 10.1089/jir.2010.0126

[70] Degrandi D, Kravets E, Konermann C, Beuter-Gunia C, Klumpers V, et al (2013) Murine Guanylate Binding Protein 2 (mGBP2) controls Toxoplasma gondii replication. Proc Natl Acad Sci U S A 110: 294–299. 23248289 10.1073/pnas.1205635110 PMC3538222

[71] Martens S, Parvanova I, Zerrahn J, Griffiths G, Schell G, et al (2005) Disruption of Toxoplasma gondii parasitophorous vacuoles by the mouse p47-resistance GTPases. PLoS Pathog 1: e24. 16304607 10.1371/journal.ppat.0010024 PMC1287907

[72] Taylor GA, Collazo CM, Yap GS, Nguyen K, Gregorio TA, et al (2000) Pathogen-specific loss of host resistance in mice lacking the IFN-gamma-inducible gene IGTP. Proc Natl Acad Sci U S A 97: 751–755. 10639151 10.1073/pnas.97.2.751 PMC15402

[73] Khan IA, MacLean JA, Lee FS, Casciotti L, DeHaan E, et al (2000) IP-10 is critical for effector T cell trafficking and host survival in Toxoplasma gondii infection. Immunity 12: 483–494. 10843381 10.1016/s1074-7613(00)80200-9

[74] Nishikawa Y, Tragoolpua K, Inoue N, Makala L, Nagasawa H, et al (2001) In the absence of endogenous gamma interferon, mice acutely infected with Neospora caninum succumb to a lethal immune response characterized by inactivation of peritoneal macrophages. Clin Diagn Lab Immunol 8: 811–816. 11427432 10.1128/CDLI.8.4.811-817.2001 PMC96148

[75] Dubey JP, Hattel AL, Lindsay DS, Topper MJ (1988) Neonatal Neospora caninum infection in dogs: isolation of the causative agent and experimental transmission. J Am Vet Med Assoc 193: 1259–1263. 3144521

[76] Hay WH, Shell LG, Lindsay DS, Dubey JP (1990) Diagnosis and treatment of Neospora caninum infection in a dog. J Am Vet Med Assoc 197: 87–89. 2370226

[77] Roos DS, Donald RG, Morrissette NS, Moulton AL (1994) Molecular tools for genetic dissection of the protozoan parasite Toxoplasma gondii. Methods Cell Biol 45: 27–63. 7707991 10.1016/s0091-679x(08)61845-2

[78] Koshy AA, Fouts AE, Lodoen MB, Alkan O, Blau HM, et al (2010) Toxoplasma secreting Cre recombinase for analysis of host-parasite interactions. Nat Methods 7: 307–309. 20208532 10.1038/nmeth.1438 PMC2850821

[79] Leng J, Butcher BA, Egan CE, Abi Abdallah DS, Denkers EY (2009) Toxoplasma gondii prevents chromatin remodeling initiated by TLR-triggered macrophage activation. J Immunol 182: 489–497. 19109180 10.4049/jimmunol.182.1.489 PMC2651083

[80] Du P, Kibbe WA, Lin SM (2008) lumi: a pipeline for processing Illumina microarray. Bioinformatics 24: 1547–1548. 18467348 10.1093/bioinformatics/btn224

[81] Smyth GK (2005) limma: Linear Models for Microarray Data. In: Gentleman R, Carey V, Huber W, Irizarry R, Dudoit S, editors. Bioinformatics and Computational Biology Solutions Using R and Bioconductor: Springer New York. pp. 397–420.

[82] Smyth GK (2004) Linear models and empirical bayes methods for assessing differential expression in microarray experiments. Stat Appl Genet Mol Biol 3: Article3. 16646809 10.2202/1544-6115.1027

[83] Reiner A, Yekutieli D, Benjamini Y (2003) Identifying differentially expressed genes using false discovery rate controlling procedures. Bioinformatics 19: 368–375. 12584122 10.1093/bioinformatics/btf877

[84] Benjamini Y, Hochberg Y (1995) Controlling the False Discovery Rate: A Practical and Powerful Approach to Multiple Testing. Journal of the Royal Statistical Society Series B (Methodological) 57: 289–300.

[85] Miller JA, Cai C, Langfelder P, Geschwind DH, Kurian SM, et al (2011) Strategies for aggregating gene expression data: the collapseRows R function. BMC Bioinformatics 12: 322. 21816037 10.1186/1471-2105-12-322 PMC3166942

[86] Dennis G Jr, Sherman BT, Hosack DA, Yang J, Gao W, et al (2003) DAVID: Database for Annotation, Visualization, and Integrated Discovery. Genome Biol 4: P3. 12734009 PMC193660

[87] Huang da W, Sherman BT, Lempicki RA (2009) Systematic and integrative analysis of large gene lists using DAVID bioinformatics resources. Nat Protoc 4: 44–57. 19131956 10.1038/nprot.2008.211

[88] Livak KJ, Schmittgen TD (2001) Analysis of relative gene expression data using real-time quantitative PCR and the 2(-Delta Delta C(T)) Method. Methods 25: 402–408. 11846609 10.1006/meth.2001.1262

