# Supplementary figures and images for: Differential Induction of TLR3-Dependent Innate Immune Signaling by Closely Related Parasite Species
Source: PLoS One. 2014 Feb 5;9(2):e88398. doi: 10.1371/journal.pone.0088398 (PMC3914978; doi:10.1371/journal.pone.0088398)

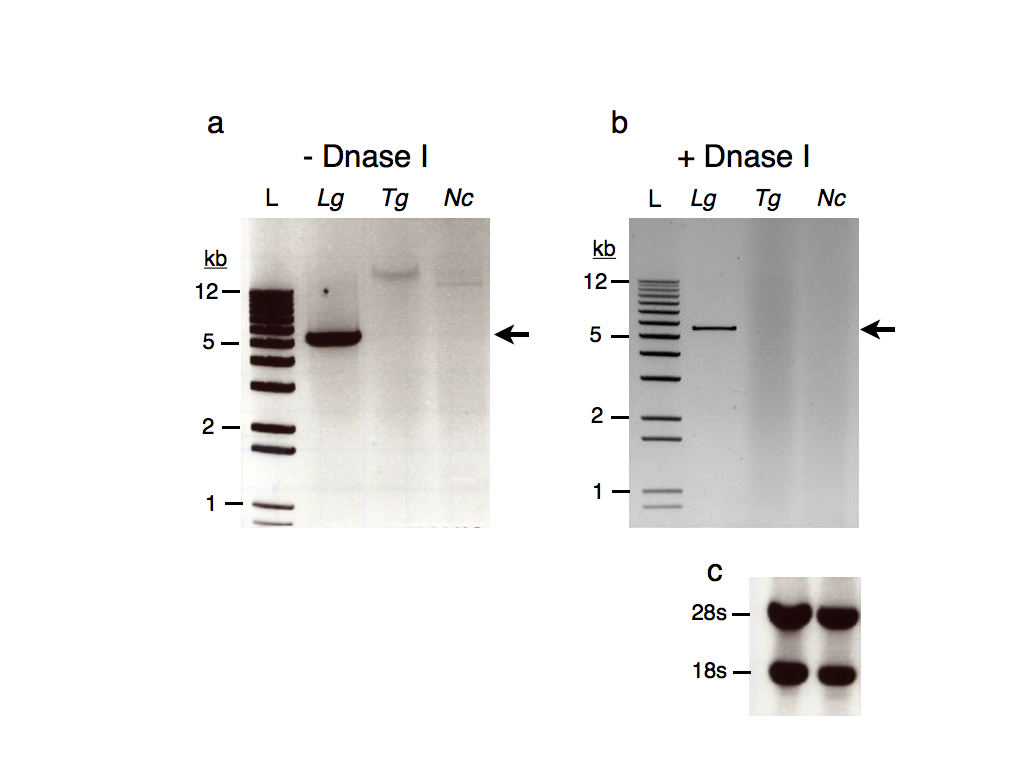

Supplement: Figure S1 — Assaying for double stranded RNA in Toxoplasma and Neospora . 0.8% agarose gel electrophoresis and ethidium bromide staining of Leishmania guyanensis (Lg), Toxoplasma gondii (Tg) and Neospora caninum (Nc) total RNAs treated with A) S1 nuclease, B) DNase I and S1 nuclease, C) untreated total RNA of Toxoplasma gondii (Tg) and Neospora caninum (Nc). 28S and 18S rRNAs bands sensitive to S1 nuclease shown as loading control. DNA ladder (L) with kilobase (kb) markers is shown. Arrow indicates an S1 nuclease- and Dnase-resistant double stranded RNA from L. guyanensis that corresponds to a known endogenous RNA virus [31]. (TIFF) [file pone.0088398.s001.tiff]

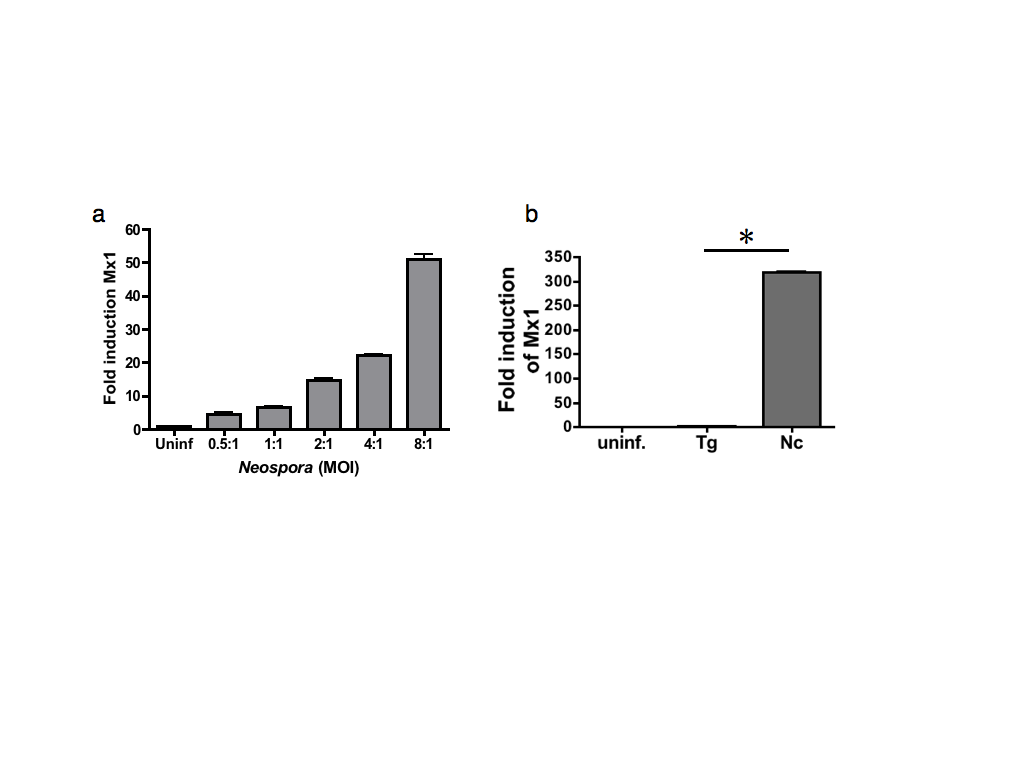

Supplement: Figure S2 — Host response to Neospora infection correlates with inoculum and is not restricted to human cells. QPCR analysis of the expression of the antiviral gene Mx1 in (a) HFF cells infected with Neospora tachyzoites at various multiplicity of infection (MOI), and (b) bovine primary fibroblasts infected with either Toxoplasma (Tg) or Neospora (Nc). Similar results were obtained for Irf7 (not shown). Error bars indicate standard deviations for two biological replicates; * = P≤0.01. (TIFF) [file pone.0088398.s002.tiff]

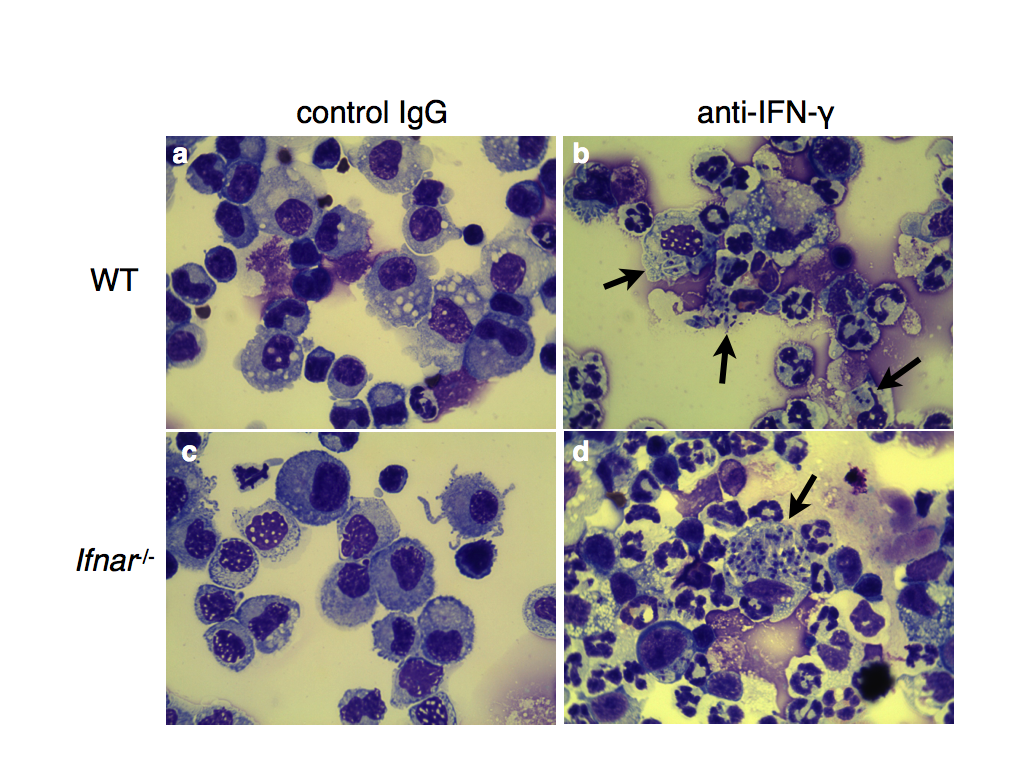

Supplement: Figure S3 — IFN-γ, but not type I interferons, are essential for control of high-dose Neospora infection in vivo . Diff-quik stained cytospins of peritoneal exudate cells recovered from (a-b) WT and (c-d) Ifnar1 -/- mice (7 days post-infection) treated with either control IgG or neutralizing antibody to IFN-γ (clone XMG-1.2). Experiments were repeated two times, with 4-5 mice per group. Representative micrographs are shown. Arrows indicate parasite-infected cells. (TIFF) [file pone.0088398.s003.tiff]
